# Pulmonary vasodilation by sildenafil in acute intermediate-high risk pulmonary embolism: a randomized explorative trial

**DOI:** 10.1186/s12890-021-01440-7

**Published:** 2021-02-28

**Authors:** Asger Andersen, Farhad Waziri, Jacob Gammelgaard Schultz, Sarah Holmboe, Søren Warberg Becker, Tage Jensen, Hanne Maare Søndergaard, Karen Kaae Dodt, Ole May, Ulrik Markus Mortensen, Won Yong Kim, Søren Mellemkjær, Jens Erik Nielsen-Kudsk

**Affiliations:** 1 Department of Cardiology, Aarhus University Hospital, Palle Juul-Jensens Boulevard 99, 8200 Aarhus N, Denmark; 2 Diagnostic Centre, Region Hospital of Silkeborg, Silkeborg, Denmark; 3 Department of Internal Medicine, Region Hospital of Randers, Randers, Denmark; 4 Department of Cardiology, Region Hospital of Viborg, Viborg, Denmark; 5 Department of Internal Medicine, Region Hospital of Horsens, Horsens, Denmark; 6 Department of Internal Medicine, Region Hospital of Herning, Herning, Denmark

**Keywords:** Pulmonary embolism, Sildenafil, PDE5 inhibition, Pulmonary vasodilation

## Abstract

**Background:**

To investigate if acute pulmonary vasodilation by sildenafil improves right ventricular function in patients with acute intermediate-high risk pulmonary embolism (PE).

**Methods:**

Single center, explorative trial. Patients with PE were randomized to a single oral dose of sildenafil 50 mg (n = 10) or placebo (n = 10) as add-on to conventional therapy. The time from hospital admission to study inclusion was 2.3 ± 0.7 days. Right ventricular function was evaluated immediately before and shortly after (0.5–1.5 h) randomization by right heart catheterization (RHC), trans-thoracic echocardiography (TTE), and cardiac magnetic resonance (CMR). The primary efficacy endpoint was cardiac index measured by CMR.

**Results:**

Patients had acute intermediate-high risk PE verified by computed tomography pulmonary angiography, systolic blood pressure of 135 ± 18 (mean ± SD) mmHg, increased right ventricular/left ventricular ratio 1.1 ± 0.09 and increased troponin T 167 ± 144 ng/L. Sildenafil treatment did not improve cardiac index compared to baseline (0.02 ± 0.36 l/min/m2, *p* = 0.89) and neither did placebo (0.00 ± 0.34 l/min/m2, *p* = 0.97). Sildenafil lowered mean arterial blood pressure (− 19 ± 10 mmHg, *p* < 0.001) which was not observed in the placebo group (0 ± 9 mmHg, *p* = 0.97).

**Conclusion:**

A single oral dose of sildenafil 50 mg did not improve cardiac index but lowered systemic blood pressure in patients with acute intermediate-high risk PE. The time from PE to intervention, a small patient sample size and low pulmonary vascular resistance are limitations of this study that should be considered when interpreting the results.

*Trial Registration*: The trial was retrospectively registered at www.clinicaltrials.gov (NCT04283240) February 2nd 2020, https://clinicaltrials.gov/ct2/show/NCT04283240?term=NCT04283240&draw=2&rank=1.

## Background

Acute pulmonary embolism (PE) is a potentially fatal disease with a broad spectrum of clinical presentations. It may vary from subtle changes in functional capacity to hemodynamic collapse and death depending on the thromboembolic mass obstructing the pulmonary circulation and the associated vasoactive response of the pulmonary arteries. In patients with high risk PE, mortality ranges from 25 to 52% and in patients with intermediate-high risk PE, mortality ranges from 8 to 15% [1, 2]. The true incidence of PE is difficult to assess, but with an estimated incidence of 6–20/10,000/year in Europe and the United States [3–5] it is a common acute cardiovascular disease.

The treatment of choice is based on the severity of hemodynamic changes and the degree of right ventricular dysfunction. Cornerstones in the treatment of PE are anticoagulation therapy in most patients and thrombolysis in hemodynamic unstable patients. Other treatment options are surgical or catheter based embolectomy or fragmentation of the thrombus mass [6–8]. Despite modern management, PE is still a fatal disease with a high risk of death in patients with intermediate or high-risk PE [6].

The ultimate cause of death in PE is right heart failure due to the acute increase in afterload [7]. This is not only due to mechanical obstruction of the proximal pulmonary arteries but also caused by active pulmonary vasoconstriction mediated by hypoxemia, a neurogenic reflex and the release of vasoconstrictors from activated lung cells, endothelial cells, platelets, and leukocytes [8–11].

As current therapies mainly focus on removing the mechanical obstruction there may be a potential for pulmonary vasodilators as add-on to current treatment strategies. Inhaled nitric oxide (NO) has shown promising results in case reports [12] and the treatment was found to be safe in a phase 1 clinical trial [13]. A recent randomized clinical trial failed at meeting its primary endpoint of normalization of echocardiogram and troponins, but there was improvement in RV function in PE patients treated with NO [14]. Administration of NO is by inhalation trough a nasal cannula or via mechanical ventilation and long-term treatment is not feasible. Pharmacological stimulation of the NO vasodilatory pathway by the oral route may be an attractive alternative to NO therapy.

NO exerts its vasodilator action by up-regulating intracellular cyclic guanosine mono phosphate (cGMP) in the smooth muscle cells of the pulmonary vasculature. cGMP is converted to the inactive 5′GMP by phosphodiesterase type 5 (PDE_5_). Hence, another strategy for pulmonary vasodilation is inhibition of the breakdown of cGMP by sildenafil, a specific PDE_5_ inhibitor widely used in the treatment of pulmonary arterial hypertension. Supporting the biologic rationale for the use of sildenafil in the treatment of PE is a series of pre-clinical studies and few case reports of its efficacy [15–17]. Furthermore, the ease of use and the low cost makes sildenafil an attractive therapy as add-on to conventional therapy. The aim of this trial was to test the acute effects on right ventricular function of pulmonary vasodilation by sildenafil administered as a single oral dose 50 mg as add-on to conventional therapy in patients with acute intermediate-high risk PE.

## Methods

### Patients

Patients with acute pulmonary embolism confirmed by contrast enhanced computed tomography (CT) were eligible for inclusion if they had symptom duration of less than 14 days, were older than 18 years and had a right ventricular/left ventricular ratio (RV/LV) > 1 measured by trans-thoracic echocardiography (TTE, 1 cm above the atrio-ventricular valves in the four-chamber view at end-diastole). Patients were excluded if they were pregnant, had cardiac arrest that required cardiopulmonary resuscitation, a life expectancy < 120 days, systolic blood pressure < 90 mmHg, metal implants, obesity or claustrophobia that excluded the patient from cardiac magnetic resonance (CMR), altered mental status making the patient unable to provide informed consent, recent use of drugs with influence on the NO-cGMP pathway, known or suspected chronic thromboembolic pulmonary hypertension, inability to perform study protocol < 72 h after conventional PE treatment was instituted or active bleeding after thrombolysis. Patients were screened for inclusion at hospitals in the Central Region of Denmark.

### Protocol

The study was designed as a single centre, prospective, randomized, placebo-controlled, double-blind, explorative trial. If eligible for inclusion, the patient was transferred to Aarhus University Hospital to complete the study protocol. To characterize the patients, RV/LV ratio was calculated from the CT scan and by TTE performed at the local institution on the day of diagnosis. Cardiac biomarkers [troponin T/I, N-terminal pro-brain natriuretic peptide (NT-proBNP)], height, weight, respiratory frequency, peripheral saturation, and pulse were measured at the local institution on the day of screening and by the study site on the day of inclusion. A detailed medical history was collected including duration of symptoms, known active cancer, recent surgery/trauma, chronic obstructive pulmonary disease, diabetes mellitus, oestrogen therapy, and/or immobilisation.

**Fig. 1:**
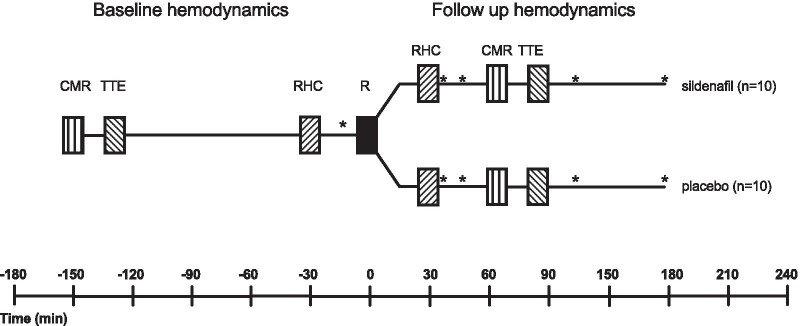
Study design. *CMR* cardiac magnetic resonance imaging. *TTE* trans-thoracic echocardiography. *RCH* right heart catheterization. *R* randomisation sildenafil 50 mg or placebo. *Measurement of blood pressure

During the study protocol (Fig. 1), right ventricular function was evaluated on the same day before and shortly after (0.5–1.5 h) randomization to sildenafil 50 (n = 10) mg or placebo (n = 10) with CMR, TTE and RHC. Primary efficacy endpoint was cardiac index (CI) evaluated by CMR. Blood pressure was evaluated at 30, 45, 120 and 180 min after randomization as a safety measure. Other hemodynamic parameters were analysed as exploratory endpoints. Exploratory endpoints evaluated by TTE were RV/LV ratio, fractional area change (FAC), tricuspid annular plane excursion (TAPSE), pulmonary artery acceleration time (PAAT) and tricuspid regurgitation gradient (TI). Exploratory endpoints evaluated by CMR were heart rate (HR), RV stroke volume (SV), RV end-diastolic volume (EDV), RV end-systolic volume (ESV), right ventricular ejection fraction (EF), mean peak longitudinal strain (MPLS). Exploratory endpoints evaluated by right heart catheterization were mean pulmonary capillary wedge pressure (mPCWP), diastolic-, systolic- and mean pulmonary artery pressure (dPAP, sPAP, mPAP), mixed venous oxygen saturation (SvO_2_) and pulmonary vascular resistance (PVR). Detailed protocols for TTE, CMR and RHC are available in the Additional file 1. Study drugs (sildenafil or placebo) were prepared in quickly absorbable gelatinous caps by the institutional pharmacy to blind both patients and the physicians to treatment. The dose of 50 mg sildenafil was chosen based on previous case reports [16, 17] where sildenafil improved hemodynamics in patients with acute PE and a study investigating acute hemodynamic effects of sildenafil in patients with pulmonary hypertension where an i.v. dose equivalent to 50 mg sildenafil effectively lowered MPAP and PVR without any hemodynamic compromise [18]. Patients were randomized in blocks of 10 with sequentially randomly numbered containers. The randomization key was disclosed by the institutional pharmacy after data analysis was complete. All analyses were performed with the observer blinded to treatment. The study was performed and data collected at Aarhus University Hospital, Denmark. The study adheres to CONSORT guidelines.

### Statistics

Sample size was calculated to be 10 patients in each treatment group. The calculation was based on the assumptions of a power of 0.9 and α of 0.05 to detect a paired difference of 29 mL/kg/min change in cardiac index with standard deviation of the difference to be 25 mL. Normality testing was performed with the Shapiro–Wilk test. Within-group comparison of continuous data was performed using the two-sided paired *t*-test. Between-group continuous data and baseline patient characteristics were compared using the two-sided unpaired Students *t*-test as an explorative analysis. Two-way ANOVA testing was performed to test differences in systemic blood pressure between groups. If data was not normally distributed, non-parametric tests were used (Wilcoxon matched pairs signed-rank or rank sum test) accordingly. All data are presented as mean ± standard deviation (SD). *P* ≤ 0.05 was considered statistically significant.

## Results

### Patients

We enrolled 21 patients from the 1st of march 2015 through 10th of September 2017 and randomly assigned 20 patients to a single 50 mg dose of sildenafil (n = 10) or placebo (n = 10). All patients randomized to treatment had acute PE verified by CT pulmonary angiography, increased RV/LV ratio measured on CT and TTE and increased cardiac biomarkers measured by troponin T/I and/or NT-proBNP. More patients had previous deep venous thromboses in the placebo group. None of the patients were treated with thrombolysis. Other baseline characteristics were similar in both groups (Table 1). Time from hospital admission and initiation of conventional therapy to study inclusion were 2.3 ± 0.7 days. Mean CI for all patients included was 2.3 ± 0.6 l/min/m^2^. CMR was performed in all patients. Inclusion rate was low initially as several patients wanted to avoid right heart catheterization (RHC). To improve inclusion rate, RHC was made optional 4 months after initiation of the study. RHC was performed in 16 patients (sildenafil n = 8 and placebo n = 8). TTE were performed in all patients, but the results from 2 patients were excluded before unblinding due to poor image quality (placebo n = 2). One patient did not have RV/LV > 1 on TTE on the day of inclusion and was excluded as screening failure before randomization.

**Table 1:** Baseline patient characteristics

	Placebo (SD)	Sildenafil (SD)	*p*-value
	n = 10	n = 10	
Age (years)	72 (9)	63 (9)	0.09
Female (%)	50	30	0.51
Height (cm)	170 (10)	171 (7)	0.87
Weight (kg)	87 (13)	86 (10)	0.83
BMI (kg/m^2^)	30 (3)	30 (4)	0.77
Known active cancer (%)	0	0	1.00
Immobilisation (%)	10	20	0.59
Recent surgery/trauma (%)	10	20	0.59
Previous VTE (%)	50	10	0.04
COPD (%)	0	20	0.17
DM (%)	30	10	0.17
Smoking (%)	10	10	1.00
Estrogen (%)	10	10	1.00
Duration of symptoms (d)	3.0 (1.3)	2.7 (1.3)	0.66
UFH/LMH/VKA/NOAK (n)	2/7/0/1	0/9/0/1	na
Oxygen therapy (l)	1.8 (1.4)	1.9 (1.9)	0.85
TnT (ng/l)	198 (131)	136 (150)	0.66
NT proBNP (ng/l)	6573 (8624)	1201 (113)	0.08
RF (/min)	18 (3)	19 (3)	0.19
Periferal saturation (%)	96 (2)	95 (2)	0.13
Pulse (BPM)	70 (9)	85 (12)	0.02
BPs (mmHg)	134 (10)	137 (24)	0.55
BPd (mmHg)	81 (9)	88 (12)	0.25
RV diameter* (cm)	5.4 (0.5)	5.2 (0.8)	0.70
LV diameter* (cm)	3.7 (0.5)	3.2 (0.5)	0.06
RV/LV*	1.5 (0.3)	1.7 (0.5)	0.23

*BMI* body mass index, *VTE* venous thrombus embolism, *COPD* chronic obstructive pulmonary disease, *DM* diabetes mellitus, *UFH* unfractioned heparin, *LMH* low molecular weight heparin, *VKA* vitamin-K antagonist, *NOAK* non-vitamin-K oral anti-coagulation, *TnT* troponin-T, *NT-proBNP* N-terminal pro brain natriuretic peptide, *RF* respiratory frequency, *BPs* systolic blood pressure, *BPd* diastolic blood pressure, *RV* righ ventricle, *LV* left ventricle, *RV/LV* right ventricular/left ventricular diameter ratio, *measured by computed tomography at the day of screening for inclusion. Data is reported as mean ± SD

### Primary and explorative endpoints for right ventricular function

**Fig. 2:**
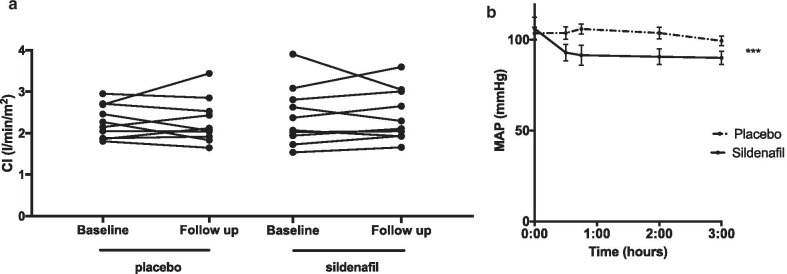
**a** Primary efficacy endpoint. *CI* cardiac index measured by cardiac magnetic resonance imaging, **b** safety endpoint. *MAP* mean arterial blood pressure. ***p < 0.001

Primary and explorative endpoints are summarized in Fig. 2a and Table 2. Time from administration of study drug to right ventricular evaluation were 32 ± 5 min for RHC, 74 ± 17 min for CMR, and 84 ± 40 min for TTE. Sildenafil did not improve cardiac index (primary endpoint) measured by CMR (Placebo baseline vs. follow up 2.28 ± 0.40 vs. 2.29 ± 0.54, *p* = 0.97; sildenafil baseline vs. follow-up 2.37 ± 0.70 vs. 2.38 ± 0.61, *p* = 0.89). Exploratory endpoints measured by CMR revealed that sildenafil significantly reduced end-diastolic and end-systolic RV volume. mPCWP increased with placebo but was unaltered by sildenafil. PAAT was shortened after placebo treatment but was unaltered by sildenafil. Other exploratory endpoints were unaltered both by sildenafil and placebo.

**Table 2:** Exploratory endpoints

	Placebo			Sildenafil		
	Baseline	Follow Up	*p*-value	Baseline	Follow up	*p*-value
HR (BPM)	69 (9)	68 (7)	0.49	84 (12)	85 (12)	0.58
* Cardiac magnetic resonance imaging derived parameters*						
RV SV (mL)	68 (19)	68 (18)	0.96	57 (19)	58 (22)	0.84
CO (l/min)	4.53 (0.94)	4.54 (1.14)	0.97	4.71 (1.44)	4.76 (1.34)	0.89
RV EDV (mL)	222 (19)	222 (12)	1.00	193 (73)	184 (76)	0.05
RV ESV (mL)	152 (18)	153 (15)	0.77	135 (62)	125 (62)	0.04
RV EF (%)	32 (8)	31 (8)	0.50	31 (8)	34 (9)	0.14
MPLS (%)	− 12.3 (3.4)	− 13.1 (3.4)	0.17	− 12.9 (4.1)	− 13.1 (4.0)	0.89
* Right heart catheterization derived parameters*						
mPCWP (mmHg)	11.1 (4.5)	12.4 (4.4)	0.02	17.0 (4.9)	15.0 (3.7)	0.14
PAPd (mmHg)	15.7 (3.2)	17.6 (2.6)	0.31	17.1 (3.1)	17.0 (6.7)	0.96
PAPs (mmHg)	47.0 (6.6)	47.4 (4.8)	0.92	47.0 (7.7)	42.9 (5.3)	0.21
mPAP (mmHg)	27.0 (2.9)	27.6 (2.4)	0.66	27.3 (3.7)	25.3 (3.4)	0.30
PVR (wood)	3.5 (1.0)	3.5 (1.4)	0.86	2.3 (1.0)	2.0 (1)	0.43
SVO_2_ (%)	62.6 (6.7)	64.4 (8.5)	0.29	65.9 (8.5)	65.2 (7.3)	0.67
* Trans thoracic echocardiography derived parameters*						
RV/LV	1.10 (0.05)	1.12 (0.19)	0.39	1.12 (0.11)	1.08 (0.21)	0.47
RV FAC (%)	0.30 (0.14)	0.30 (0.11)	0.98	0.34 (0.15)	0.37 (0.14)	0.28
TAPSE (cm)	1.79 (0.44)	1.78 (0.56)	0.86	1.69 (0.36)	1.79 (0.44)	0.33
PAAT (ms)	64 (12)	54 (12)	0.02	61 (8)	64 (13)	0.40
TI (mmHg)	36 (8)	36 (10)	0.81	33 (8)	31 (3)	0.52

Exploratory endpoints assesed by heartrate (HR), cardiac magnetic resonance derived paramters, right heart catheterization derived parameters and transthoracic echocardiograhy derived parameters.  * RV SV* right ventricular stroke volume, *CO* cardiac output, *RV EDV* right ventricular end diastolic volume, *RV ESV* right ventricular end systolic volume, *RV EF* right ventricular ejection fraction, *MPLS* mean peak longitudinal strain, *mPCWP* mean pulmonary capillary wedge pressure, *PAPd* diastolic pulmonary artery pressure, *PAPs* systolic pulmonary artery pressure, *mPAP* mean pulmonary artery pressure, *PVR* pulmonary vascular resistance, *SVO*_*2*_ mixed venous oxygen saturation, *RV/LV* right ventricular/left ventricular diameter ratio, *RV FAC* right ventricular fractional area change, *TAPSE* tricuspid annular plane systolic excursion, *PAAT* pulmonary artery acceleration time. TI tricuspid regurgitation gradient. Data is reported as mean ± SD

### Safety

None of the patients reported adverse events. Blood pressure was lower in sildenafil treated patients compared to placebo (Fig. 2b). The maximal drop in blood pressure was observed 120 min after administration of study drug (sildenafil: -19 ± 10, placebo: 0 ± 9 mmHg, *p* = 0.0007) to a MAP of 91 ± 14 mmHg in the sildenafil and 104 ± 10 in the placebo treated patients. The largest drop in systemic blood pressure in a single patient was from 146/95 to 94/64 mmHg observed 180 min after sildenafil treatment.

## Discussion

Pulmonary vasodilation by sildenafil have shown benefit in experimental models of acute pulmonary embolism and in case reports. This is the first randomized controlled trial to test sildenafil in patients with acute intermediate-high risk pulmonary embolism.

In a previous trial, epoprostenol failed to show benefit on the RV in patients with acute PE. However, patients in that trial had only slight RV dysfunction making it difficult to demonstrate a hemodynamic benefit of pulmonary vasodilatation [24]. In the current trial, all patients had an increased RV/LV ratio and an increase in cardiac biomarkers. With a blood pressure > 90 mmHg the patients are characterized as intermediate-high risk PE according to current guidelines from the European Society of Cardiology [20]. Cardiac index measured by CMR was lower than healthy individuals and similar to patients with congestive heart failure in NYHA class III–IV [21]. Also, mPAP was increased compared to healthy individuals [22]. Therefore, it is unlikely that patient selection with regards to the severity of the disease can explain the overall neutral result of this trial.

We used several independent methods to evaluate RV function. Previous and ongoing studies on a similar topic have used RV/LV ratio measured by echo as a primary endpoint [22, 23]. By the use of a more elaborate RV evaluation in this study we could potentially measure subtle hemodynamic changes even with a low number of patients. In the exploratory endpoints, we did find a reduction in EDV and ESV. With an unaltered PVR, these changes could simply be secondary to the observed lower systemic blood pressure which emphasizes the accuracy of the hemodynamic evaluation.

We chose to test the acute effects of pulmonary vasodilation after administration of a single dose of sildenafil 50 mg as this design has previously proven to be effective in PAH patients [23] and in preclinical studies of PE [19]. The observation of a lowered MAP at several independent timepoints after administration similar to previous observations [23] confirm that sildenafil was administered in an effective dose and that the effect was evaluated at relevant timepoints after administration.

The magnitude of MAP lowering together with the observation that none of the patients reported adverse events suggest that sildenafil was safe in our patients. It should however be emphasized that data is limited and we cannot conclude that sildenafil is generally safe in acute PE based on our data alone.

## Limitations

There are some limitations to our study. One is the delay of 2.3 days from initiation of conventional therapy to study inclusion. It has been suggested that vasoconstriction is more pronounced in the early phase after PE and not so much in the later phase [24, 25]. Hence, the potential effect of pulmonary vasodilation in acute PE would be blunted at study inclusion more than 2.3 days after hospital admission. There are limited clinical data supporting this speculation but as experimental data suggest, the efficacy of pulmonary vasodilation is attenuated during the first 12 h in the initial phase after PE [26] which could explain the discrepancy between the positive experimental studies that initiate pulmonary vasodilation shortly after PE and the few, but all neutral clinical studies where onset of treatment is delayed.

We observed a higher than expected left atrial pressure yielding a near-normal PVR. This finding could explain the overall neutral result of this trial, as sildenafil is most effective in treating pre-capillary pulmonary vasoconstriction [27]. It should, however be taken into consideration that the patients did have a high thrombus burden increasing the risk of over-wedging and a possible false high wedged pressure.

Another limitation is the low number of patients included in the trial. Being an exploratory study, we choose an elaborate hemodynamic evaluation of the patients to identify possible hemodynamic changes. Post-hoc power calculation revealed that 3924 patients should be included to show a significant difference with a power of 0.8 and α of 0.05 to detect a paired difference of the observed 17 mL/m^2^/min change in cardiac index using the observed standard deviation of 380 mL/m^2^/min. Looking at Fig. 2 however, there is one sildenafil treated patient with a CI of 3.8 L/m^2^/min at baseline and as discussed previously there would be rationale behind excluding patients with a high cardiac output in future trials. If this was done in this trial with a cut-off of CI < 3 L/m^2^/min, a non-significant improvement of 112 mL/m^2^/min was observed (*p* = 0.2). Although speculative, this observation could be of use in future trials. A post hoc power calculation for the more widely used RV/LV ratio as primary endpoint using the observed 0.04 change in RV/LV ratio with SD of 0.16 reveals that 128 patients should be included to show an effect in the treatment arm with a power of 0.8 and α of 0.05 suggesting that RV/LV ratio may be a more robust primary endpoint in future trials. The calculations above discuss statistical effect. Whether or not these small improvements in hemodynamics would translate into a clinical effect is unknown.

The limitations in this trial should be taken into consideration in the interpretation of the data. There is substantial experimental evidence in a wide range of PE models that pulmonary vasodilation improve hemodynamics in acute PE(10) but still, there are only a very limited number of small clinical trials. Therefore, it would still be relevant to explore the clinical potential of pulmonary vasodilation in acute PE. Patient selection with regards to high RV/LV ratio, high PVR, low CI and timing will probably be key to success in future trials.

## Conclusion

In conclusion we found that a single dose of sildenafil 50 mg lowered systemic blood pressure but did not improve cardiac index in patients with acute intermediate-high risk pulmonary embolism. The time from PE to intervention, a small patient sample size and low pulmonary vascular resistance are limitations of this study that should be considered when interpreting the results.

## Supplementary Information

**Additional file 1.** Supplementary methods for acuisition and analysis of magnetic resonance imaging, trans thoracic echocardiography, and right heart catheterization.

## Data Availability

The datasets used and analysed during the current study are available from the corresponding author on reasonable request. Protocol and detailed methods and materials are available in the Additional file 1.

## Abbreviations

PE: Pulmonary embolism
RHC: Right heart catheterization
TTE: Trans thoracic echocardiography
CMR: Cardiac magnetic resonance imaging
NO: Nitric oxide
cGMP: Cyclic guanosine mono phosphate
PDE_5_: Phosphodiesterase type 5
CT: Contrast enhanced computed tomography
RV/LV: Right ventricular/left ventricular ratio
NT-proBNP: N-terminal pro-brain natriuretic peptide
CI: Cardiac index
FAC: Fractional area change
TAPSE: Tricuspid annular plane excursion
PAAT: Pulmonary artery acceleration time
TI: Tricuspid regurgitation gradient
HR: Heart rate
SV: Stroke volume
EDV: RV end-diastolic volume
ESV: RV end-systolic volume
EF: Right ventricular ejection fraction
MPLS: Mean peak longitudinal strain
dPAP: Diastolic pulmonary artery pressure
sPAP: Systolic pulmonary artery pressure
mPAP: Mean pulmonary artery pressure
SvO_2_: Mixed venous oxygen saturation
PVR: Pulmonary vascular resistance
SD: Standard deviation
