# Supplementary material for: Pulmonary vasodilation by sildenafil in acute intermediate-high risk pulmonary embolism: a randomized explorative trial
Source: BMC Pulm Med. 2021 Feb 28;21:72. doi: 10.1186/s12890-021-01440-7 (PMC7916297; doi:10.1186/s12890-021-01440-7)
Supplement: Supplementary file 1 — Additional file 1. Supplementary methods for acuisition and analysis of magnetic resonance imaging, trans thoracic echocardiography, and right heart catheterization. [file 12890_2021_1440_MOESM1_ESM.docx]

# Supplementary

### 12.1 Acquisition and analysis of MRI data

MRI was conducted using a Philips Ingenia dStream 1.5 T whole body MR scanner (Philips Medical Systems, Best, Netherlands). The cardiac rhythm was monitored with an integrated 4-electrode electrocardiogram (ECG) system during the entire session. The ECG was used to synchronize data acquisition. Cine scanning (time-resolved imaging) was used to assess the right ventricular function and right and left ventricuar geometry. A survey scan was performed followed by an ECG-triggered temporally resolved cine using a balanced steady-state free precession sequence during breath-hold. Pulmonary flow measurements were obtained using a free-breathing, ECG-triggered phase contrast sequence. Image parameters for the cine scans were: slice thickness 8 mm, slice gap 0 mm, repetition time/echo time/flip angle 3.1 ms/1.55 ms/60°, 350 × 350 mm field of view, 176 × 153 acquisition matrix, and 30 phases within one cardiac cycle. A stack of 3 long-axis slices were acquired for 4-chamber view, and for short-axis view a stack of 10-12 slices were acquired covering the entire right and left ventricle. The parameters for the phase contrast sequence were: echo time (TE) = 6.1 ms, repetition time (TR) = 9.3 ms, phase percentage = 107 %, field of view (FOV) = 300 mm × 255 mm, matrix = 128 × 82, number of phases = 50, number of excitations (NEX) = 2, and slice thickness (ST) = 7 mm. To avoid aliasing, encoding velocity was set to 150 cm/s.

Image analysis was performed with the observer blinded to the source of the sample using Segment v2.2 R6246. Right ventricular volumes were measured by manually tracing the endocardium at end-diastole and end-systole on all slices encompassing the heart. Right ventricular ejection fraction was calculated by dividing end systolic- with end diastolic volumes. Pulmonary flow curves were obtained by drawing a region of interest at the cross-sectioned pulmonary artery in the phase-contrast scan. Stroke volume and cardiac output were calculated using heart rates recorded simultaneously.

### 12.2 Acquisition and analysis of echocardiographic data

Echocardiography was performed with standard available equipment at our department: Vivid s6, Vivid E95, Vivid E90 (GE healthcare, Horton, Norway) according to guidelines from the American society of echocardiography.{Rudski:2010it}Analysis of TTE images were performed off-line using Echo Pac software (v110, GE , Horton, Norway) with the observer blinded to the source of the sample. Right-to-left ventricular dimension (RV/LV) was measure 1 cm above the atrio-ventricular plane. Fractional area change was analyzed by measuring the right ventricle end-systolic (RV-ESA) and end-diastolic (RV-EDA) area in the four chamber view and computing it as: FAC = (RV-EDA-RV-ESA)/RV-EDA x 100. Tricuspid annular plane excursion (TAPSE) was obtained by placing a m-mode cursor through the RV apex and the lateral tricuspid annulus. Then, longitudinal displacement was measured from peak-diastole to peak-systole. Pulmonary artery acceleration time (PAAT) was measured from the initiation to the peak of the systolic PW Doppler curve obtained in the right ventricular outflow tract. The Doppler-determined peak systolic pressure gradient was with the modified Bernoulli equation using GE software on the peak CW Doppler curves of the tricuspid regurgitation jet.

### 12.3 Acquisition and analysis of right heart catheterization data

Right heart catheterization was done with a triple lumen Swan-Ganz thermistor and balloon-tipped catheter introduced into the jugular vein and advanced to the pulmonary artery guided by fluoroscopy. Mean pulmonary capillary wedge pressure (mPCWP) was measured from the tip after wedging the catheter with an inflatable balloon in a smaller pulmonary artery and measuring the pressure distal to the ballon. Systolic, diastolic and mean pulmonary artery pressure (PAP) was measured in the pulmonary artery with the catheter. Pulmonary vascular resistance (PVR) was computed using PVR = (mPAP-mPCWP)/CO. Mixed venous saturation (SVO_2_) was obtained by blood gas analysis of blood obtained from the pulmonary.
